# Marriage and Scientific Selection

**Published:** 1905-09-23

**Authors:** 


### MARRIAGE AND SCIENTIFIC SELECTION.

In a paper devoted to "The Delay of Old Age and the Alleviation of Senility," Dr. Charles Stockton[1] bewails the prevailing disregard by civilised peoples of the well-recognised laws of inheritance. He points out that a proper and systematic study of the subject is rendered almost impossible by the absence of records as to family tendencies in bulk. Records of estates are common but of family peculiarities little can be learnt, a state of affairs which leads the author to the wish that some kind of official record might be instituted to throw light upon the strains of men, that is to say upon the physical and mental proclivities of different stocks. "It is not improbable," he says, "that if such records were known to exist they would not only be consulted, but would gradually grow to have an influence on the formation of families. Love is said to know no barriers, but history fails to confirm this view. If a matter of class, or caste, or rank, has been sufficiently important to control the forming of marriage contracts, it is not impossible to conceive of a more intelligent public sentiment which would make an alliance with a family celebrated for intellectual, physical, and moral superority a thing greatly to be desired; and conversely, which would lead to the abhorrence of an alliance with a family in which freaks, degenerates and incompetents, or in which a tendency towards lowered nutrition, too early maturity or premature senility, were frequent events."

There is much food for reflection in the sociological problems underlying heredity, and no thoughtful member of our profession can dismiss lightly the weight of trouble laid upon innocent lives by the almost criminal carelessness with which society flouts the elementary rules of wisdom in the contracting of marriages. These rules of wisdom are as old at least as the Utopia of Sir Thomas More. "In choosing their wives" he says, "they use a method that would appear to us very absurd and ridiculous, but it is constantly observed among them, and is accounted perfectly consistent with wisdom. Before marriage some grave matron presents the bride, naked, to the bridegroom, and after that some grave man presents the bridegroom, naked, to the bride. We, indeed, both laughed at this and condemned it as very indecent. But they on the other hand wondered at the folly of the men of all other nations, who, if they are but to buy a horse of small value, are so cautious that they will see every part of him, and take off both his saddle and all his other tackle, that there may be no secret ulcer hid under any of them; and that yet in the choice of a wife, on which depends the happiness or unhappiness of the rest of his life, a man should venture upon trust, and only see about a handsbreadth of the face, all the rest of the body being covered, under which may lie hid what may be contagious as well as loathsome."

The interest of this reference is of course academic, and the suggestion covers but a fraction of the ground. The well-being of any two given members of the community may be admitted to be a matter of importance, but it is insignificant in comparison with the well-being of their progeny in proportion as these exceed their parents in number. Moreover it is quite obvious that a mere cursory physical examination by an interested party is an idle suggestion, for some of the most dreaded stigmata of degenerate inheritance are not revealed by any physical characteristics.

We have dealt with these suggestions more seriously than their present impracticability perhaps deserves, because there is underlying them a principle which the medical profession cannot teach too assiduously—the principle, namely, of the responsibility of parentage. In the absence of a properly educated public opinion on this subject, legal enactments, such as have been attempted in some parts of the United States, are foredoomed to failure and the scandals of irregular unions. As Dr. Stockton observes, we have to cultivate a public demand that none but healthy parents shall be allowed to continue the race, for no man has a right to beget disease. He points out that no effort is spared to interfere with the course of nature by rescuing the unfit among us from the elimination prescribed for them, and that society can claim in return the right to veto the propagation of degeneracy by such individuals. There is no need to despair, for signs are not wanting that among educated people questions of heredity are allowed some weight in the contracting of marriages, though the tendency towards the concealment of family taints discounts well-intentioned efforts to a considerable extent. It has been wisely said that no parent should permit a child to marry until the prospective bride or bridegroom can produce from a reputable insurance company an acceptance of her or his life at ordinary rates. Here is a ready means to hand of determining fitness; its adoption would no doubt increase the number of runaway matches to some extent, but it would help to give pause to hasty and emotional people, and might be expected to reduce the sum of human misery to an extent undreamt of by its unwitting authors.

[1] Med. Rec. (N.Y.), July 15, 1905.

